# A modified Yamane technique with a posterior approach in a case report of intraocular lens dislocation

**DOI:** 10.1016/j.ijscr.2025.111369

**Published:** 2025-04-25

**Authors:** Ludovico Iannetti, Carmen Baratta, Flavia Figliola, Marta Armentano, Giacomo Visioli, Ludovico Alisi

**Affiliations:** a Ophthalmology Unit, Head and Neck Department, Policlinico Umberto I University Hospital, Sapienza University of Rome, Viale del Policlinico 155, 00161 Rome, Italy; b Department of Sense Organs, Sapienza University of Rome, Piazzale Aldo Moro 5, 00185 Rome, Italy

**Keywords:** Yamane technique, Scleral fixation, PMMA loops, Dislocated IOL, Vitreoretinal surgery

## Abstract

**Introduction:**

The Yamane intrascleral fixation technique is a widespread surgical approach used in ophthalmology to secure three-piece intraocular lenses (IOLs) without the use of sutures. This technique is particularly beneficial in cases where the capsular bag is not suitable for IOL implantation. By creating a flange at the tip of each haptic this procedure innovatively eliminates the need for additional securing materials. We report an alternative management of posterior intraocular lens (IOL) dislocation with a modified Yamane technique, performed in vitreous chamber.

**Case presentation:**

We describe the surgery of a 56-year-old male with posterior dislocation of the capsular bag and a three-piece IOL with Polymethylmethacrylate (PMMA) loops into the vitreous cavity. His medical history included retinal detachment treated with scleral buckling 40 years earlier and cataract surgery performed 4 years earlier. The patient underwent a 25-gauge pars plana vitrectomy. Scleral tunnels were created to externalize the PMMA loops and securing the IOL in a stable position according to the classic Yamane technique: a bimanual approach with a 30-gauge x 13 mm ultra-thin-walled needle and maxgrip forceps was used.

**Discussion:**

Modified Yamane technique performed in vitreous chamber, especially in selected cases of dislocated IOL with PMMA loops, may allow better handling of the IOL and the loop insertion. This modified technique, by reducing mechanical stress on the PMMA loops, contributes to long-term IOL integrity and lowers the risk of complications during surgery.

**Conclusion:**

This technique may offer a reliable alternative for ophthalmic surgeons managing PMMA IOLs dislocations.

## Highlights

• Management of posterior IOL dislocation with a modified approach of the Yamane technique.
• Chandelier light ensures adequate posterior lighting, facilitating bimanual maneuvers.
• Procedure conducted directly in the vitreous chamber.
• This technique improves surgical outcomes and maintains the IOL's structural integrity.

## 1 Introduction

The Yamane intrascleral fixation technique is a widespread surgical approach used in ophthalmology to secure three-piece intraocular lenses (IOLs) without the use of sutures. This technique is particularly beneficial in cases where the capsular bag is not suitable for IOL implantation [1]. The procedure was described by Dr. Shin Yamane et al. [2] in 2017 and consists of a flanged intrascleral intraocular lens fixation with a double-needle. Traditionally, sutures or surgical glue have been used to achieve this fixation [3,4]. This technique innovatively eliminates the need for additional securing materials by creating a flange at the tip of each haptic. Additionally, it does not require scleral fixation sutures, which are often used in other methods to secure the IOL. By eliminating these sutures, the procedure reduces potential complications and the likelihood of suture-related issues over time such as postoperative infections, inflammation, irritation and increased IOP [5,6].

The traditional Yamane technique begins with two angled incisions parallel to the limbus, made using a 30-gauge thin-wall needle; the precise angle is crucial for proper IOL positioning and stability. The haptics are then gently externalized with needle guidance. Cauterization creates a bulbous flange at the tip of each haptic, which is then secured within the scleral tunnels. Primarily performed in the anterior segment, this technique ensures lens centration and stability, ultimately optimizing visual outcomes. As with any surgical procedure, expertise and technical precision are essential to minimize risks and achieve optimal results [2]. This technique was initially reported using both polymethylmethacrylate (PMMA) and polyvinylidene fluoride (PVDF) loops. However, the latter material, being more flexible, theoretically could provide an easier approach [7].

In this communication, we report an alternative management of posterior IOL dislocation with a modified approach of the Yamane technique, performed directly in the vitreous cavity. This work is reported in line with the SCARE 2023 criteria [8].

## 2 Case report

A 56-year-old myopic male presented with a posterior IOL-capsular bag dislocation into the vitreous cavity in the left eye (LE) and pre-operative BCVA of 1.1 LogMar. The patient reported a retinal detachment in the LE treated 40 years before with scleral buckling and a cataract surgery 4 years before, with a three-piece IOL implantation. The specific IOL used was an Alcon MN60MA +5D (Alcon, Fort Worth, Texas USA), with PMMA loops.

**Fig. 1:**
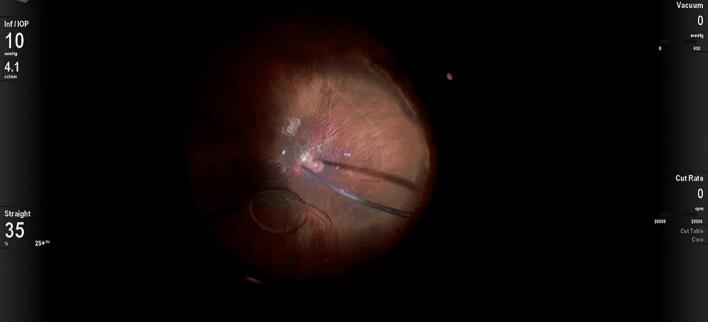
Injection of PFCL to protect the posterior pole and to move the IOL anteriorly.

**Fig. 2:**
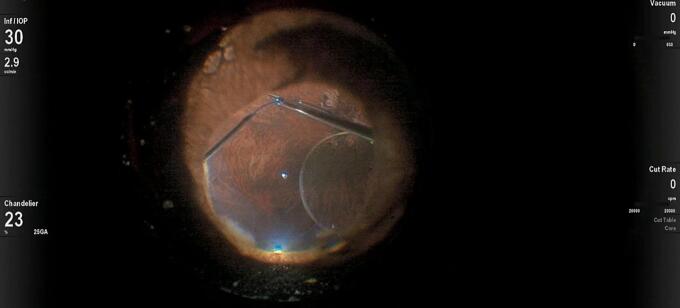
The first PMMA loop of the IOL was captured with maxgrip forceps and guided into the lumen of the 30-gauge thin-walled needle.

**Fig. 3:**
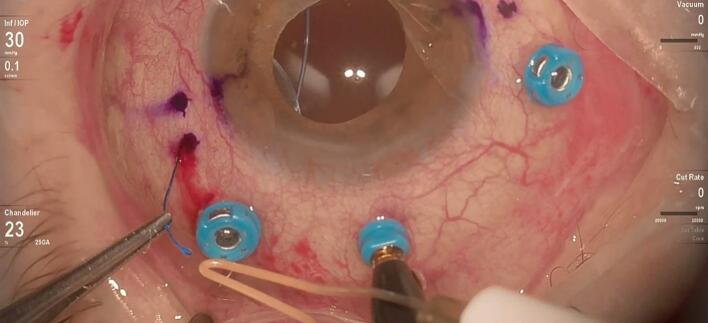
Loop extracted from the sclera and its terminal end melted with low-temperature cautery to form a bulb at the 3 o'clock position.

**Fig. 4:**
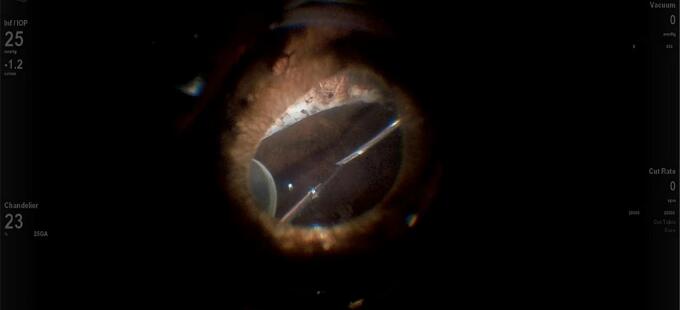
The second PMMA loop of the IOL was captured with maxgrip forceps and guided into the lumen of the 30-gauge thin-walled needle.

**Fig. 5:**
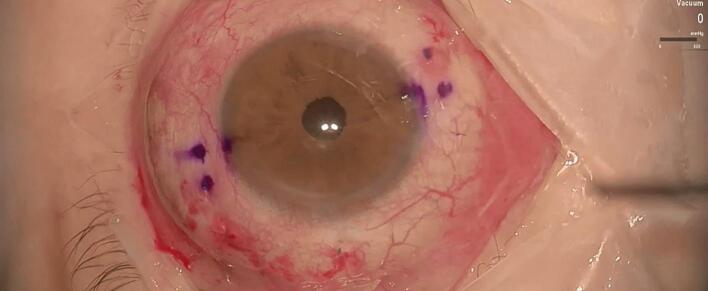
IOL well positioned and centered at the end of surgery.

A 25-gauge Pars Plana Vitrectomy (PPV) was performed with Alcon CONSTELLATION Vision System (Alcon, Fort Worth, Texas USA), Alcon NGENUITY 3D (Alcon, Fort Worth, Texas USA), and Leica RUV800 (Leica microsystems, Wetzlar, Germany). A chandelier light was used to ensure adequate posterior lighting. The procedure began with an anterior vitrectomy, followed by the removal of the capsular bag. Subsequently, both central and peripheral vitrectomies were performed. Perfluorocarbon liquid (PFCL) was then injected to protect the posterior pole, effectively displacing the IOL anteriorly and away from the retinal plane (Fig. 1). A distance of 2.5 mm from the sclero-corneal limbus was measured at the 3 o'clock and 9 o'clock positions using a compass, to create a scleral tunnel. The identified points were then labeled with a conjunctival marker. A PRC-300131 30 Gauge × 13 mm ultra-thin-walled needle (TSK STERiJECT, TSK Laboratory, Japan) was then folded to a 70° angle and inserted into the vitreous chamber, creating a 2.5 mm long transconjunctival scleral tunnel at the 3 o'clock position. Subsequently, the procedure was conducted directly in the vitreous chamber. The first PMMA loop of the IOL was captured with maxgrip forceps and gently guided into the lumen of the thin-walled needle (Fig. 2). The loop was then extracted from the sclera, and its terminal end was melted with low-temperature cautery to form a bulb (Fig. 3). The haptic was subsequently extruded through the conjunctiva and secured into the scleral tunnel. The same maneuver was repeated with the second loop at the 9 o'clock position, exactly 180° across from the first sclerotomy (Fig. 4). The PFCL was then removed from the vitreous cavity. The centering of the IOL was verified, and the chandelier light and trocars were removed (Fig. 5). The patient was examined at intervals of 1 day, 7 days, 1 month, and 3 months following the surgery. The recovery was uneventful, with no complications observed. The final BCVA was 0.3 LogMar. No significant tilting or dislocation of the IOL was noticed at any timepoint with slit lamp examination.

## 3 Discussion

A sutureless Yamane intrascleral fixation technique performed through a posterior approach provides several significant benefits, particularly in the context of managing IOLs. One of the foremost advantages is the ability to reposition a dislocated IOL in a minimally invasive manner. In fact, the approach we present minimizes the risk of iris contact and reduces the IOL manipulation compared to the classic Yamane approach [1,2]. Compared to the traditional technique, this procedure eliminates the necessity for corneal incisions, and it is applicable particularly when the IOL in question is a three-piece lens with PMMA loops [6,9].

The modified Yamane technique with a posterior approach has already been described by Tansu Erakgun [10], however our case presents some differences from his case. Erakgun [10] performed a 23-gauge PPV, while we performed a 25-gauge PPV, and he used a 27-gauge needle to insert the haptics of the IOL, while we used an ultrathin 30-gauge needle, consistent with the classic Yamane technique [1,2,10]. Further, when the maxgrip forceps grabs the second loop of the IOL it could interfere with the already inserted first IOL loop. Thus, it is essential to adequately place the trocars in a proper position, preferably at 11 o’clock and 1 o’clock, further from the needle’s sclerotomy incision, to avoid this eventuality.

This modified Yamane technique, when applied with a posterior approach in the vitreous chamber, offers significant advantages in specific cases, particularly in the management of IOLs with PMMA loops. This approach can improve the handling of the IOL and the insertion of the loops, reducing some of the limitations encountered with the classic Yamane technique [1,2].

Unlike PVDF loops, PMMA loops are more prone to stress-related damage during manipulation. The posterior approach of the modified technique can reduce these risks significantly. The vitreous cavity is larger than the anterior chamber, providing more space to maneuver the IOL and loops. This facilitates a more controlled and less stressful insertion process, reducing the potential for loop distortion or breakage, a common risk with PMMA loops. While PVDF loops are generally favored, the modified Yamane technique with a posterior approach can make PMMA loops a viable option in specific cases. Specifically, our technique is recommended in cases where a PMMA IOL is already dislocated deeply within the vitreous chamber. Moreover, filling the vitreous cavity halfway with PFCL can enhance the procedure's success. PFCL assists in elevating the IOL, which improves visibility and access during surgery. This elevation allows surgeons to better manipulate the IOL and loops, ensuring precise placement and reducing the likelihood of intraoperative complications such as loops breakage or inadvertent retinal damage. In fact, the main limitations for this technique are the potential risks of impacting the retina with the PMMA loops during the surgical maneuvers.

## 4 Conclusions

The modified Yamane technique performed in vitreous cavity could provide better performance for ophthalmic surgeons managing challenging IOL dislocations. Surgeons should consider this alternative technique that does not require corneal incisions, especially in cases of dislocated IOLs with PMMA loops, to reduce postoperative astigmatism and maintain the IOL's long-term structural integrity.

## CRediT authorship contribution statement

**Ludovico Iannetti**: Writing – review & editing, Investigation, Data curation, Conceptualization, Supervision.

**Carmen Baratta**: Writing – original draft, Data curation.

**Flavia Figliola**: Writing – original draft, Data curation.

**Marta Armentano**: Writing – review & editing, Methodology.

**Giacomo Visioli**: Writing – review & editing, Methodology.

**Ludovico Alisi**: Writing – review & editing, Methodology.

## Informed consent

Written informed consent was obtained from the patient for publication of this case report and accompanying images. A copy of the written consent is available for review by the Editor-in-Chief of this journal on request.

## Ethical approval

As a case report, the study is exempt from ethnical approval form our institution Sapienza – University of Rome.

## Ethics statement

Not applicable, as our institution does not require ethics board approval for case reports.

## Guarantor

Ludovico Iannetti

## Funding sources

This research did not receive any specific grant from funding agencies in the public, commercial, or not-for-profit sectors.

## Registration of research studies

1.Name of the registry: N/A.

2.Unique identifying number or registration ID: N/A.

3.Hyperlink to your specific registration (must be publicly accessible and will be checked): N/A.

## Declaration of competing interest

The authors declare that they have no known competing financial interests or personal relationships that could have appeared to influence the work reported in this paper.

## Data Availability

The data used to support the findings of this study are included in the article. Additional data are available upon reasonable request.

## Appendix A Supplementary data

Video 1 Posterior Yamane Video 1

Supplementary material Image 1
